# Histopathological Diagnosis of Malignant Melanoma at the Dawn of 2023: Knowledge Gained and New Challenges

**DOI:** 10.3390/dermatopathology10010013

**Published:** 2023-02-13

**Authors:** Gerardo Cazzato

**Affiliations:** Section of Molecular Pathology, Department of Precision and Regenerative Medicine and Ionian Area (DiMePRe-J), University of Bari “Aldo Moro”, 70124 Bari, Italy; gerardo.cazzato@uniba.it; Tel.: +39-3405203641

Year after year, the incidence and prevalence rates of cutaneous Malignant Melanoma (MM) show a continuous increase and, according to the most up-to-date American Cancer Society (ACS) projections, it is estimated that 97,610 new melanomas will be diagnosed in 2023 (about 58,120 in men and 39,490 in women) and approximately 7990 people are expected to die of melanoma (about 5420 men and 2570 women) [1]. The histopathologic diagnosis of MM always continues to be a cornerstone for the correct setting of the diagnostic, therapeutic, and care pathway of the affected patient, and a correct and comprehensive diagnosis accompanied by all essential information is necessary [2]. Traditionally, MM has been the “great mimic” of dermatopathology, as it can mimic the most disparate neoplasms; over time, careful research in the field of ancillary immunohistochemistry techniques has enabled the detection of antigens capable of corroborating histologic morphology alone [3]. Melan-A [4], HMB-45 [5], MiTF [6], Tyrosinase [7], and, recently, PRAME [8] help dermatopathologists on a daily basis in diagnosing atypical pigmented lesions, providing an indispensable morphological analysis, with information that can identify a lesion "melanoma" or not. 

Unfortunately, it is not that simple, because, even in the face of lesions framed with adequate immunohistochemistry, it is possible to find contradictions rather than fail to obtain inter-observer agreement, as immunostaining for melanocytic markers is also susceptible to interpretation by the pathologist’s eye, as well as potential harbingers of incorrect indications if not properly evaluated and considered. For example, as much as it is known that HMB-45 is a marker of human melanocyte immaturity/dysplasia, and we would thus be inclined to consider it as evidence of lesion of concern, in fact, there are cases (such as blue nevus with its variants and Spitz lineage nevi) that can express it while exhibiting no morpho/phenotypic features of atypia [9]. 

In the latest edition of the World Health Organization of Skin Tumours (2018), a reference is made to the nine pathways of MM, seven of which are related to cutaneous melanoma, going on to provide not only an immuno-morphological view but also molecular biology data that are indispensable in some cases of melanocytic lesion. For example, it is now clear that there is no clear-cut boundary of benign vs. malignant in melanocytic pathology, but these are different lesions with different biology and behaviors depending on the specific genetic "signature" present [10,11]. On the other hand, it is important to consider how, given the high number of melanocytic lesions that are excised and, therefore, brought to pathology laboratories, it would be unthinkable and unsustainable to perform full immunohistochemical screening as well as molecular investigations to all lesions. Therefore, morphology continues to be fundamental, which, although it cannot be the only diagnostic element capable of resolving the totality of cases, can certainly direct the pathologist to specific and further useful inquiries to confirm/deny the diagnostic hypothesis. For example, as pointed out by Ferrara G. et al., a morphology reminiscent of a Spitz nevus does not always correspond to a specific Spitz lineage, as Spitz-like morphologic aspects may not be accompanied by as many expressions of immunomarkers, which is consistent with that working hypothesis [11]. In this field and for what has already been said, it remains certain that the gold standard of malignant melanoma diagnostics remains to be histopathology and, therefore, by extension, the cultural, experiential, and cognitive background of the dermatopathologist. Therefore, we believe that only a careful and thorough knowledge of the subject is the source of a “well made” diagnosis.
